# Independent contributions of structural and functional connectivity: Evidence from a stroke model

**DOI:** 10.1162/netn_a_00207

**Published:** 2021-11-30

**Authors:** Lynsey M. Keator, Grigori Yourganov, Alexandra Basilakos, Argye E. Hillis, Gregory Hickok, Leonardo Bonilha, Christopher Rorden, Julius Fridriksson

**Affiliations:** Department of Communication Sciences and Disorders, University of South Carolina, Columbia, SC, USA; Department of Psychology, University of South Carolina, Columbia, SC, USA; Department of Communication Sciences and Disorders, University of South Carolina, Columbia, SC, USA; Department of Neurology, Johns Hopkins University School of Medicine, Baltimore, MD, USA; Department of Physical Medicine and Rehabilitation, Johns Hopkins School of Medicine, Baltimore, MD, USA; Department of Cognitive Science, Johns Hopkins University, Baltimore, MD, USA; Department of Cognitive Sciences, Department of Language Science, University of California, Irvine, CA, USA; Department of Neurology, Medical University of South Carolina, Charleston, SC, USA; Department of Psychology, University of South Carolina, Columbia, SC, USA; McCausland Center for Brain Imaging, University of South Carolina, Columbia, SC, USA; Department of Communication Sciences and Disorders, University of South Carolina, Columbia, SC, USA; McCausland Center for Brain Imaging, University of South Carolina, Columbia, SC, USA

**Keywords:** Resting-state functional connectivity, Stroke, Language, Aphasia

## Abstract

Altered functional connectivity is related to severity of language impairment in poststroke aphasia. However, it is not clear whether this finding specifically reflects loss of functional coherence, or more generally, is related to decreased structural connectivity due to cortical necrosis. The aim of the current study was to investigate this issue by factoring out structural connectivity from functional connectivity measures and then relating the residual data to language performance poststroke. Ninety-seven participants with a history of stroke were assessed using language impairment measures (*Auditory Verbal Comprehension* and *Spontaneous Speech* scores from the Western Aphasia Battery–Revised) and MRI (structural, diffusion tensor imaging, and resting-state functional connectivity). We analyzed the association between functional connectivity and language and controlled for multiple potential neuroanatomical confounders, namely structural connectivity. We identified functional connections within the left hemisphere ventral stream where decreased functional connectivity, independent of structural connectivity, was associated with speech comprehension impairment. These connections exist in frontotemporal and temporoparietal regions. Our results suggest poor speech comprehension in aphasia is at least partially caused by loss of cortical synchrony in a left hemisphere ventral stream network and is not only reflective of localized necrosis or structural connectivity.

## INTRODUCTION

It is commonly understood that anatomical white matter fiber tracts serve as the backbone of functional connectivity and shape endogenous network coherence (Adachi et al., 2012; Damoiseaux & Greicius, 2009; Goni et al., 2014; Honey & Sporns, 2008; Miŝic et al., 2016). Direct structural pathways tend to yield stronger functional connections while weaker functional connectivity is reflected in indirect anatomical connections that traverse additional regions (Goni et al., 2014). Strong blood oxygen level–dependent (BOLD) signals in the absence of direct structural pathways (Koch et al., 2002; Skudlarski et al., 2008) provide evidence that functional connectivity depends not only on direct, but also on indirect structural pathways. Additional evidence for an indirect relationship between these measures comes from reports of alterations in functional connectivity networks despite intact structural connectivity (Carter et al., 2010; He et al., 2007). The exact relationship between structural and functional connectivity, particularly in the context of a stroke-induced lesion, remains unclear.

Following brain injury, cortical infarcts result in distal disruption of structural, functional, and metabolic networks (Carrera & Tononi, 2014; Corbetta et al., 2005). The effects of structural and functional connectivity have been investigated in stroke patients (Zhang et al., 2017) and other clinical populations such epilepsy (Chiang et al., 2015; Zhang et al., 2011). Generally, lesion-induced disruptions in structural and functional connectivity lead to reduced network coherence and behavioral impairments (Carter et al., 2010; He et al., 2007; Park et al., 2011; Siegel et al., 2016; Tang et al., 2016), including aphasia, a language disorder that typically results from damage to the left hemisphere. In poststroke aphasia, network alterations yield deficits across language domains (Baldassarre et al., 2019; Klingbeil et al., 2019), and the extent of interruption to left hemisphere structural and functional connectivity networks is related to the severity of language impairment (Bonilha et al., 2014b; Butler et al., 2014; Griffis et al., 2020). To improve rehabilitation and prognostics of poststroke aphasia, it is important to determine the extent to which stroke disrupts structural and functional network architecture and, subsequently, how these changes affect language recovery.

Resting-state functional connectivity (rsFC) is a measure of functional connectivity and gauges temporal coherence of intrinsic BOLD signal fluctuations between gray matter regions (Biswal et al., 1995; Fox & Raichle, 2007). Recent work suggests functional connectivity disruption secondary to a stroke lesion may reflect damage to direct structural pathways (Griffis et al., 2020). To better understand the effects of rsFC poststroke and the nature of the relationship between structural and rsFC, many have investigated neural synchrony (measured by rsFC) to determine its role as biomarker in stroke recovery. This has been investigated across a variety of behavioral domains including language (Griffis et al., 2017; Klingbeil et al., 2019) and motor function (Arun et al., 2020; Min et al., 2020).

Setting the standard for rsfMRI in stroke patients, Siegel and colleagues (2016) published a large sample resting-state stroke study investigating disruptions of network connectivity across a variety of behavioral domains. While others have investigated the effects of rsFC in aphasia recovery, the results vary considerably (see Klingbeil et al., 2019, for a review). While disturbances to functional connectivity networks are associated with clinical deficits (Baldassarre et al., 2016; Klingbeil et al., 2019), previous investigations have not accounted for potentially confounding effects of structural connectivity. Therefore, the results cannot be solely attributed to impaired functional connectivity as they likely also reflect disruptions in structural connectivity secondary to cortical necrosis. This limitation emphasizes the poorly understood relationship between structural and functional connectivity, especially in the context of cortical damage and clinical populations such as people with aphasia.

In the current study, we investigated the relationship between functional and structural pathways and aimed to determine if temporal coherence (measured by rsFC) predicts language function poststroke after accounting for damage to white matter connections between cortical regions. To do this, we investigated a cohort of stroke patients with left hemisphere damage and considered commonly impaired language domains: speech comprehension and speech production. Curating a highly selective cohort of participants enabled us to compare data in both the damaged left hemisphere and preserved right hemispheres.

We investigated our research questions and hypotheses in the context of the dual-stream model of speech processing (Hickok & Poeppel, 2004, 2007), which suggests speech comprehension is supported by an expansive bilateral cortical network that encodes and maps speech sound to meaning and speech production relies on a dorsal stream to integrate auditory and motor function (Friederici & Wartenburger, 2010; Hickok & Poeppel, 2007; Kümmerer et al., 2013; Saur et al., 2008). Investigating a behavioral task that relies on bilateral processing (speech comprehension) allowed us to compare and contrast data from cortical regions (defined by Fridriksson et al., 2016) that were either potentially damaged left hemisphere or preserved right hemisphere. To determine if our results were reflected in other subdomains of language, we examined speech production as a secondary dependent factor. The dual-stream model suggests that speech production relies on unilateral dorsal processing pathway in the left hemisphere (Hickok & Poeppel, 2007).

Our research questions were as follows: (a) Does stroke-related speech comprehension impairment correlate with rsFC in the bilateral ventral language stream regions of interest (Hickok & Poeppel, 2004, 2007)?; (b) Does speech production correlate with rsFC in left hemisphere dorsal stream regions of interest?; (c) Does rsFC explain either speech comprehension or speech production scores after controlling for overall lesion volume and impaired structural connectivity? We hypothesized that speech comprehension scores would linearly associate with rsFC (increased functional connectivity leads to improved comprehension scores) in bilateral ventral stream regions and that bilateral ventral stream rsFC would predict behavioral scores, even after accounting for lesion volume and white matter damage. With respect to speech production, we hypothesized that due to the left lateralized brain damage in our cohort of participants, functional connectivity in the left dorsal stream would predict speech production.

## METHODS

### Participants

A cohort of 97 chronic stroke participants (40 women, mean age at stroke = 56.01 ± 11.83 years) was assessed using the Western Aphasia Battery–Revised (WAB-R) (Kertesz, 2007) to determine aphasia type and severity. All participants had incurred a left hemisphere stroke and were at least 1-year poststroke (mean months poststroke = 54.64, 1st quartile = 14, 3rd quartile = 80, interquartile range = 66) at the time of assessment. The National Institute of Health Stroke Scale (Brott et al., 1989) was assessed at baseline to all participants to quantify stroke severity (mean = 6.02 ± 3.81). This research was approved by the Institutional Review Boards at the University of South Carolina and Medical University of South Carolina and was carried out in accordance with the Declaration of Helsinki.

### Behavioral Evaluation

In accordance with the WAB-R manual, an Aphasia Quotient (a global measure of aphasia severity on a scale of 0–100, where a score below 93.8 indicates aphasia [0–25 typically indicates a very severe aphasia, 26–50 severe aphasia, 51–75 is moderate aphasia, and ≥ 76 suggests a mild aphasia]; Kertesz, 2007) was calculated using scores for each of the following subdomains: *Spontaneous Speech*, *Auditory Verbal Comprehension*, *Repetition*, and *Naming and Word Finding* for each participant (mean = 62.65 ± 24.92). Aphasia types, as classified by the WAB-R, were as follows: 41 Broca’s, 14 Conduction, 3 Wernicke’s, 5 Global, 22 Anomia, 2 Transcortical motor, and 10 individuals did not have aphasia per the WAB-R criteria. Participants without aphasia were included in the study because they suffered a left hemisphere stroke, and although they did not have chronic language deficits, they further inform the effect of stroke-induced lesions on rsFC.

To test our hypotheses, we selected behavioral tasks that primarily tax speech comprehension abilities with relatively little speech production demands. These tasks included multiple measures from the WAB-R: (a) *Yes/No Questions*, a 60-point subtest that requires participants to respond “yes” or “no” to questions of increasing complexity (including biographical content, orientation, and abstraction); (b) *Auditory Word Recognition*, a 60-point subtest where participants are asked to point to a picture or object that corresponds to a verbal stimulus (targets presented along with five distractors); and (c) *Sequential Commands*, an 80-point subtest where participants are verbally presented with sentence-level commands of increasing complexity. These three subtests constitute the *Auditory Verbal Comprehension* score, a 0–10-point-weighted scale calculated from the total of all three subtests (number of points out of 200) divided by 20. This *Auditory Verbal Comprehension* score is included as the dependent factor in the analyses described below.

To determine if our results are reflected in other subdomains of language, we examined speech production as a secondary dependent factor. For this purpose, we included participant scores from the *Spontaneous Speech* subtest of the WAB-R. The *Spontaneous Speech* score is calculated by adding WAB-R subscores: *Information Content* and *Fluency*, each of which is rated on a 10-point scale based on participants’ responses to five biographical questions and description of a picture.

### Imaging Data

#### Data acquisition.

MRI scanning was performed within 2 days of language testing. Images were acquired on Siemens 3T scanners that were upgraded from a Trio (12-channel head coil) to a Prisma (20-channel head/neck coil) at the University of South Carolina or at the Medical University of South Carolina. Structural (T1, T2), diffusion tensor imaging, and resting-state fMRI scans were acquired. Parameters were as follows:

***T1-weighted images***: 3D MP-RAGE sequence with 1-mm^3^ isotropic voxels, a 256 × 256 matrix size, 256 × 256 field of view (FOV), a 9-degree flip angle and 192 slice sequence, TR = 2,250 ms, TE = 4.11 ms, TI = 925 ms, echo time = 4.11 ms with parallel imaging (GRAPPA = 2, 80 reference lines).

***T2-weighted images***: utilized a sampling perfection with application optimized contrasts using a different flip angle evolution (3D-SPACE) sequence with 1-mm^3^ voxels; TR = 3,200 ms, TE = 567 ms, variable flip angle, 256 × 256 matrix scan with 176 slices (1 mm thick), using parallel imaging (GRAPPA = 2, 80 reference lines). This series was acquired with the same slice center and angulation as the T1-weighted sequence.

Diffusion tensor imaging using a Prisma in four series, a pair with b = 1,000 s/mm^2^ (43 volumes of which 7 were b = 0, TR = 5,250 ms, TE = 80.0 ms) and a pair with b = 2,000 s/mm^2^ (56 volumes of which 6 were b = 0, TR = 5,470 ms, TE = 85.4 ms, TA = 5:23). The pairs were identical except for reversed phase encoding polarity (A > P vs. P > A). Scans used a monopolar sequence with a 140 × 140 matrix, 210 × 210 mm FOV, multiband ×2, 6/8 partial Fourier, 80 contiguous 1.5-mm axial slices.

***Resting-state fMRI***: EPI sequence was acquired with 216 × 216 mm FOV, 90 × 90 matrix size, and a 72-degree flip angle, 50 axial slices (2 mm thick with 20% gap yielding 2.4 mm between slice centers), TR = 1,650 ms, TE = 35 ms, GRAPPA = 2, multiband ×2, sequential descending acquisition. A total of 427 volumes were acquired.

#### Preprocessing of structural scans.

**Figure 1.**
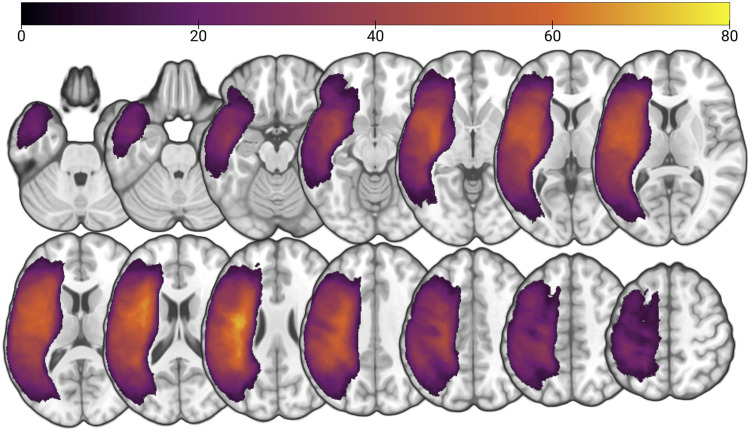
Lesion overlap map for study participants (*n* = 97). The color scale indicates the number of participants with lesion damage at a particular location. The upper boundary (*n* = 80) of the color scale indicates regions at least 80 participants had damage.

All image processing used scripts we developed and publicly share (https://github.com/neurolabusc/nii_preprocess). Lesions were manually demarcated in MRIcron (Rorden et al., 2013; Rorden & Brett, 2000) on the T2-weighted image by a neurologist (L. Bonilha) blinded to the participant’s language scores. The T2 image was coregistered to the T1 image, and T1 parameters were used to reslice the lesion into the native T1 space. Resliced lesion maps were smoothed with a 3-mm full-width half maximum Gaussian kernel to remove jagged edges associated with manual drawing and subsequently binarized using a 50% cutoff (so lesions were not dilated or eroded). Enantiomorphic normalization of the T1 scan was performed using the clinical toolbox (Rorden et al., 2013). See lesion overlay map (Figure 1).

The diffusion image was aligned with the lesion map (T2-weighted image coregistered into the T1-weighted image) and linearly normalized to the nondiffusion image (B0 image) using FSL (FMRIB Software Library) FMRIB (Functional MRI of the Brain) Linear Image Registration Tool. We used the resulting spatial transform to register probabilistic maps of white and gray matter in native T1 space and the stroke lesion into the diffusion MRI space. This yielded spatial normalization of the atlas regions of interest (ROIs) to diffusion space. The preprocessing for the current study is consistent with previous methods from our group (Yourganov et al., 2016).

#### Preprocessing of resting-state functional connectivity.

The resting-state fMRI data were corrected for motion using the SPM12 “realign and unwarp” procedure with default settings. We performed brain extraction using the SPM12 script pm_brain_mask with default settings. The mean fMRI volume for each participant was aligned to the corresponding T2-weighted image to compute the spatial transformation between the fMRI data and the lesion mask. The fMRI data were then spatially smoothed with a Gaussian kernel with full width at half maximum = 6 mm. The voxel-wise fMRI time courses were detrended using the following regressors: mean signals from the white matter and from cerebrospinal fluid; time courses of the six motion parameters estimated at the motion correction step; linear, quadratic, and cubic trends. Then, the time courses were band-pass filtered using the 0.01–0.1 Hz frequency band.

To remove artifacts driven by lesions, the procedure described by Yourganov et al. (2018) was used. Work by our group suggests this is an effective and robust method to account for cerebrospinal fluid (Yourganov et al., 2018). The FSL MELODIC package was used to decompose data into independent components, and we computed the *z*-scored spatial maps for each independent component. The spatial maps were then thresholded at *p* < 0.05 and compared with the lesion mask for that participant. If the spatial overlap (measured with Jacard index) between the lesion mask and the thresholded independent components map was greater than 5%, the corresponding component was deemed to be significantly overlapping with the lesion mask. All such components were regressed out of the fMRI data using the fsl_regfilt script from the FSL package. Additional methodological details can be found in Yourganov et al. (2018).

After these steps, individual rsFC connectomes were built for each participant by (a) segmentation of probabilistic gray matter maps from T1-weighted images; (b) division of gray matter map into ROIs based on the AICHA atlas (Joliot et al., 2015); and (c) computation of ROI-specific time courses of the BOLD signal by averaging time courses across the voxels within each ROI. Functional connectivity for a pair of ROIs was computed as bivariate Pearson’s correlation coefficient between their mean BOLD fMRI time courses.

#### Brain parcellation.

To reduce dimensionality of our data, we used 108 ROIs from the AICHA atlas (Joliot et al., 2015) to divide gray matter into ROIs. These ROIs were derived from those implicated as dorsal or ventral ROIs by Fridriksson and colleagues (2016), which relied on segmentation from the JHU atlas to identify dual-stream regions in a cohort of stroke patients (Faria et al., 2012). It is important to note that the dual-stream model proposed by Hickok and Poeppel (2004) is theoretical, so for the purposes of the current investigation, we relied on ROIs from the aforementioned Fridriksson et al. (2016) study. While there is not a 1:1 relationship between these ROIs and the proposed dual-stream model, the correspondence between the dual-stream model and the ROIs reported by Fridriksson et al. is very high.

The AICHA ROIs used in the current study included 26 ROIs in the dorsal stream (left hemisphere only) and 82 bilateral ROIs in the ventral stream (41 per hemisphere). ROIs from the AICHA atlas were implemented for the current study because this atlas separates gray matter into smaller spatial subdivisions compared to the JHU atlas, yielding better spatial sensitivity and regional specificity for the analyses. To understand the associations between damage and language performance, we characterized brain damage by using both lesion and connectome data.

### Data Analyses

#### Lesion-symptom mapping.

To understand the effects of lesion volume and location on language production and comprehension, we conducted a region-based lesion-symptom mapping analysis. All univariate statistical analyses were implemented using NiiStat toolbox for MATLAB (https://www.nitrc.org/projects/niistat/). We used 108 dual-stream ROIs (as described in “Brain Parcellation”) and controlled for total lesion volume. Only voxels where at least 10 individuals had damaged were included in the analysis. The univariate analyses used conventional lesion-symptom mapping: general linear model (GLM) with *p* < 0.05 and control for multiple comparisons used permutation thresholding (5,000 permutations).

#### Structural connectivity.

Probabilistic tractography was applied to evaluate pairwise gray matter structural connectivity (Bonilha et al., 2015; Gleichgerrcht et al., 2017). Tractography was estimated using the FMRIB Diffusion Toolbox probabilistic method (Behrens et al., 2007) with FDT’s BEDPOST (default parameters: 3 fibers per voxel, ARD weight of 1, burn-in period of 1,000, 1,250 jumps, and one sample every 25) to build default distributions of diffusion parameters at each voxel. Structural connectivity was qualified as fiber count between the aforementioned 108 ROIs (the number of streamlines arriving in one region when another ROI was seeded and vice versa). All possible connections between nodes were included, without any a priori constraints regarding plausibility, with the subsequent analyses identifying those where connectivity strength was reliably associated with behavioral impairment. Univariate analyses consistent with those used for lesion-symptom mapping were performed. All analyses controlled for total lesion volume and corrected for multiple comparisons using permutation analyses (5,000 permutations).

#### Resting-state functional connectivity.

NiiStat (https://github.com/neurolabusc/NiiStat) was used to analyze the association between rsFC within dual-stream ROIs and performance on the behavioral subtests for auditory comprehension and spontaneous speech. To examine the association between rsFC across ROIs and each WAB-R subdomain score (*Auditory Verbal Comprehension* and *Spontaneous Speech*), we computed a GLM where behavioral scores served as dependent variables and functional connectivity scores between each pair of ROIs were the independent variables. Values for each predictor (*p* < 0.05) were *z*-transformed using SPM’s smp_t2z function. The statistical threshold for all analyses was corrected for multiple comparisons by using permutation analyses (5,000 permutations).

**Figure 2.**
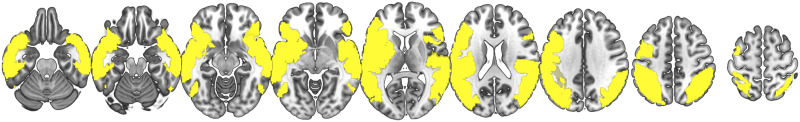
AICHA dual-stream ROIs. Dual-stream ROIs as defined by the AICHA atlas (Joliot et al., 2015). ROIs, regions of interest.

To address our first two research questions, we considered the relationship between temporal coherence (as defined by rsFC) and comprehension and production scores in dual-stream ROIs (108 regions, as described in section 1.3.4; see Figure 2). To do this, *Spontaneous Speech* and *Auditory Verbal Comprehension* scores from the WAB-R were included as dependent variables in the GLM and total lesion volume was included as a confound variable.

To address the third research question and determine if rsFC poststroke explains language scores after controlling for total lesion volume and impaired structural connectivity, we controlled for total lesion volume by regressing it out of behavioral scores and controlled for structural connectivity by regressing out the fiber count between each pair of ROIs for the same pair’s rsFC. In sum, to account for both lesion volume and structural pathways, these variables were included as a nuisance regressors in the model. Notably, only *Auditory Verbal Comprehension* scores were included as a dependent variable as no significant predictors were identified for *Spontaneous Speech* scores in the previous analysis.

In a final analysis of rsFC, we included one dependent variable (*Auditory Verbal Comprehension* scores) and a third anatomical covariate, which we qualified as damage to temporoparietal “critical areas.” These critical areas were identified in the previous functional connectivity analyses, suggesting coherence between these regions is a statistically significant predictor of language impairment scores. We calculated the lesion load (mm^3^) to the left hemisphere angular gyrus, inferior parietal gyrus, middle temporal gyrus, and left inferior temporal gyrus and summed these values to yield total lesion volume in “critical areas.” We used this analysis to confirm that previous rsFC analyses were not simply reflective of damage to respective cortical regions and instead provide additional information unique from that which is contained in the lesion data alone.

**Figure 3.**
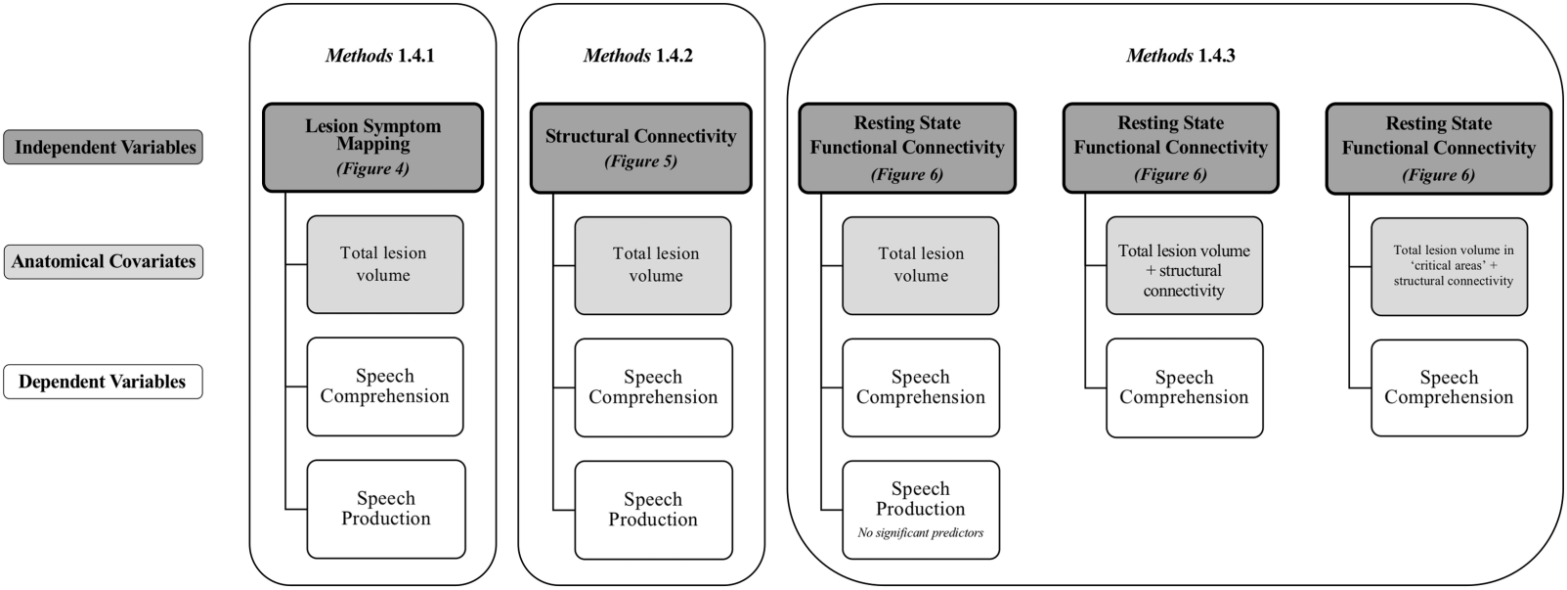
Flow chart of neuroimaging analyses. Neuroimaging modalities, anatomical covariates, and dependent variables described in Methods 1.4.1–1.4.3 are outlined in the current figure. Speech comprehension is quantified as *Auditory Verbal Comprehension* scores and speech production as *Spontaneous Speech* scores (WAB-R). Figure numbers correspond to the results from each analysis.

In summary, three resting-state functional connectivity analyses were conducted, each incorporating different anatomical covariates: (a) total lesion volume, (b) total lesion volume and structural connectivity (DTI), and (c) lesion load to “critical areas” and structural connectivity (DTI). For the first analysis, *Auditory Verbal Comprehension* scores (a measure of speech comprehension) and *Spontaneous Speech* (a measure of speech production) were included as dependent variables in the GLM. For the second two analyses, only *Auditory Verbal Comprehension* scores were included as a dependent variable as no significant predictors for *Spontaneous Speech* scores survived the first rsFC analysis. See Figure 3 for an outline of analyses.

### Data Availability

Data are available from authors by request.

## RESULTS

### Lesion-Symptom Mapping

**Figure 4.**
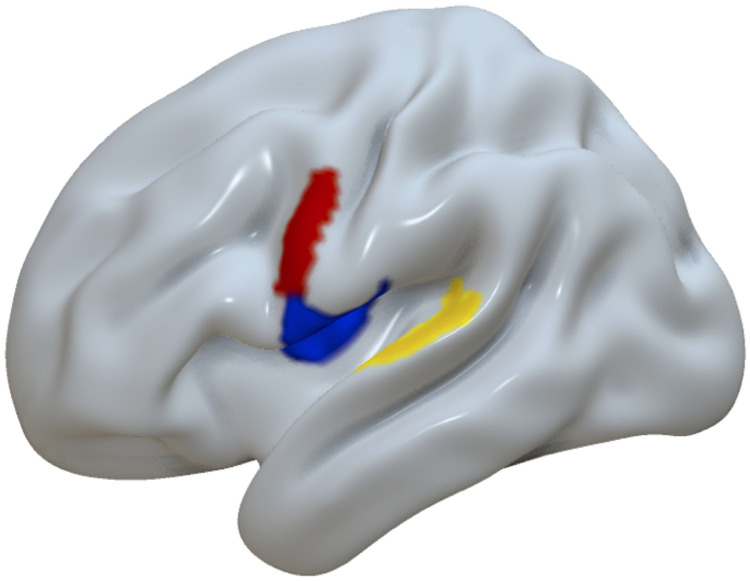
Lesion-symptom mapping analysis for speech performance. Damage to the STG was associated with lower speech comprehension scores (yellow, *z* = −2.97); damage to the precentral gyrus was associated with higher comprehension scores (red, *z* = 3.09). Damage to the Rolandic operculum was associated with poorer *Spontaneous Speech* scores (blue, *z* −2.92 and −2.97).

A region-based lesion-symptom mapping analysis (controlling for total lesion volume) revealed that damage to the superior temporal gyrus (STG, *z* = 2.97) was associated with lower speech comprehension scores, whereas damage to the precentral gyrus was associated with higher speech comprehension scores (*z* = 3.09; Figure 4). Damage to two frontal regions of the Rolandic operculum (*z* = −2.92 and = −2.97) was negatively correlated with speech production scores (Figure 4).

### Structural Connectivity

**Figure 5.**
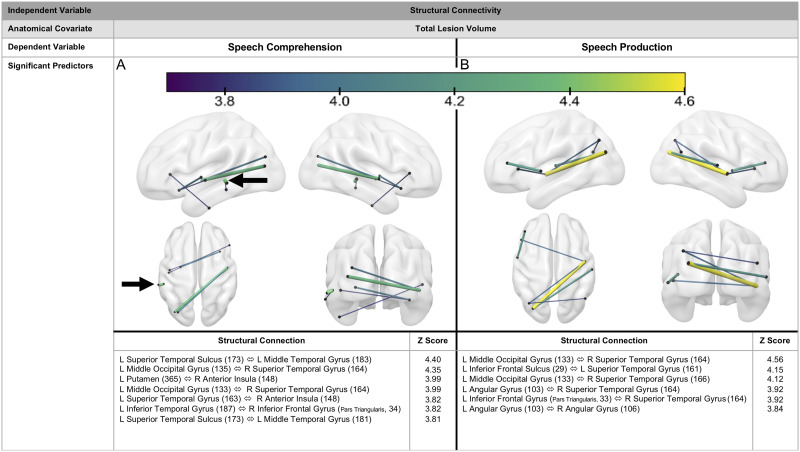
Structural connectivity predicting language scores. Seven structural pathways, two left hemisphere and five interhemispheric connections, predicted speech comprehension scores, after controlling for total lesion volume. The strongest predictor of speech comprehension (L superior temporal sulcus to L middle temporal gyrus, *z* = 4.40) is indicated with a black arrow (A). Six white matter pathways predict speech production scores, after controlling for total lesion volume (B). Color lines indicate positive *z*-scores (i.e., greater structural connectivity is associated with better language scores from the WAB-R). *Z*-scores indicate strength of association with speech comprehension scores. Warmer colors and thicker edges indicate higher *z*-scores. WAB-R = Western Aphasia Battery–Revised. Numbers in parentheses indicate subregions of ROIs, as designated by the AICHA atlas (Joliot et al., 2015).

Inter- and intrahemispheric structural pathways predicted speech comprehension and speech production scores. Integrity of seven structural pathways predicted speech comprehension (*z* = 3.81–4.40, Figure 5A). The strongest predictor of speech comprehension scores was a left hemisphere temporal pathway between the left superior temporal sulcus and left middle temporal gyrus. No right hemisphere intrahemispheric structural pathways significantly predicted behavior. Six structural pathways predicted speech production (*z* = 3.84–4.56, Figure 5B). *Spontaneous Speech* scores were best predicted by interhemispheric pathways with the exception of one left hemisphere frontotemporal connection from the inferior frontal sulcus to STG (*z* = 4.15).

### Resting-State Functional Connectivity

#### Regressing total lesion volume.

**Figure 6.**
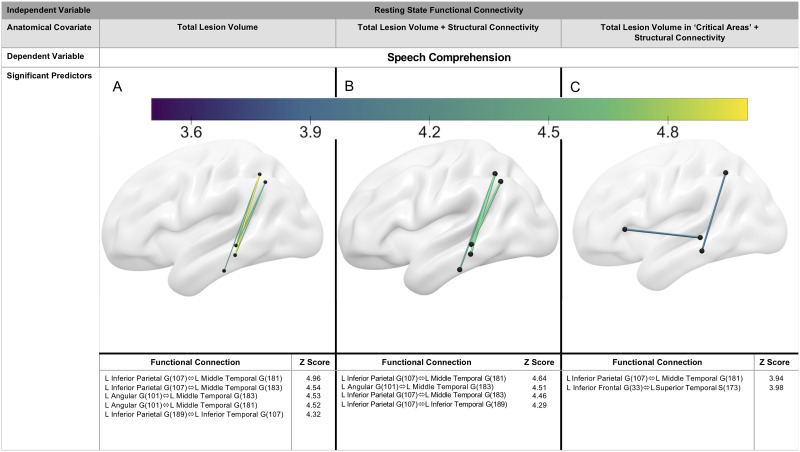
Resting-state functional connectivity predicting speech comprehension. (A) Five temporoparietal connections in the left hemisphere ventral stream predicted speech comprehension, after controlling for total lesion volume. (B) Four temporoparietal connections in the left hemisphere ventral stream predicted speech comprehension scores, after controlling for total lesion volume and structural connectivity. (C) Two resting-state connections in the left hemisphere ventral stream predicted speech comprehension, after accounting for lesion load in temporoparietal ROIs and structural connectivity. *Z*-scores indicate strength of association with *Auditory Verbal Comprehension* scores. Colored lines indicate positive *z*-scores (i.e., greater temporal coherence is associated with better *Auditory Verbal Comprehension* scores from the WAB-R). Warmer colors and thicker edges indicate higher *z*-scores. Numbers in parentheses indicate subregions of ROIs, as designated by the AICHA atlas (Joliot et al., 2015).

After controlling for total lesion volume, five temporoparietal functional connections, exclusive to the left ventral stream, predicted speech comprehension scores (*z* = 4.32–4.96, see Figure 6A). Preserved rsFC in these left hemisphere ventral stream regions were associated with better speech comprehension performance. There were no right hemisphere intrahemispheric nor interhemispheric connections that significantly predicted speech comprehension. No connections survived statistical thresholding for predicting speech production.

#### Regressing lesion volume and structural connectivity.

After controlling for total lesion volume and structural connectivity, four temporoparietal functional connections remained statistically significant predictors of speech comprehension scores. These included all but one of the functional connections identified in (controlling for total lesion volume, but not structural connectivity), albeit with lower overall *z*-scores (*z* = 4.29–4.64, see Figure 6B).

#### Regressing lesion volume to ‘critical areas’ and structural connectivity.

A final resting-state functional connectivity analysis of left hemisphere ventral stream ROIs was conducted to confirm that the results described in “Regressing total lesion volume” and “regressing lesion volume and structural connectivity” do not simply reflect frank damage to temporoparietal cortical regions. After regressing out lesion volume in temporoparietal areas and fiber counts between these regions, two resting-state functional connections in the left hemisphere ventral stream predicted speech comprehension performance: (a) left inferior parietal gyrus to left middle temporal gyrus and (b) left inferior frontal gyrus to left superior temporal sulcus (see Figure 6C). The connection between the inferior parietal gyrus and middle temporal gyrus (*z* = 3.94) was consistent with our results above.

## DISCUSSION

### Summary of Findings

The present study investigated rsFC in the chronic stage of stroke recovery. We aimed to determine (a) if speech comprehension performance was associated with rsFC in bilateral ventral streams, (b) if speech production scores correlated with rsFC in the dorsal stream, and (c) if rsFC predicted comprehension or production scores after controlling for the potentially confounding effects of stroke-induced cortical necrosis and impaired structural connectivity.

Relying on data from a cohort of chronic stroke participants, we provide evidence that neural synchrony (as measured by rsFC) in the left ventral stream predicts speech comprehension performance. In an initial lesion-symptom mapping analysis, our findings are consistent with the literature that supports posterior damage results in impaired verbal comprehension while anterior damage causes impaired language expression. These results suggest suprasylvian damage tends to be countercorrelated with infrasylvian damage. Therefore, patients with more precentral gyrus damage tend to have less STG damage. This result does not suggest patients with damage to precentral regions can understand better, but rather that they are less likely to have temporal lobe damage, specifically damage to the STG. These initial lesion mapping results served as quality control and informed subsequent analyses to determine the effects of such lesion on structural and functional connectivity.

We identified temporoparietal functional connections where decreased neural coherence is associated with impaired speech comprehension, namely, connections between the angular gyrus and middle/inferior temporal regions. Even after accounting for cortical damage and structural connectivity in these regions, neural coherence in the left ventral stream specifically, between frontal and temporal regions, explained speech comprehension scores highlighting the predictive power of residual rsFC in language performance. Such findings confirm that coherence in the left hemisphere ventral stream provides predictive information beyond that contained in the lesion data. Contrary to our hypothesis, no significant associations between resting-state functional connectivity and language scores were identified in the right hemisphere, nor were resting-state functional connections in either hemisphere associated with *Spontaneous Speech* scores. This is addressed further in the *Limitations* section.

### Network Changes Poststroke

The current study expands upon network-based studies from our group and others that have implemented whole-brain connectivity mapping to understand network effects of stroke-induced necrosis. It is well understood that expansive structural and functional networks support language (Corbetta et al., 2018; Hope et al., 2017; Salvalaggio et al., 2020; Siegel et al., 2016; Stockert et al., 2016), and frank cortical damage affects overall network connectivity (Cramer, 2008; Kreisel et al., 2006; Nudo & Friel, 1999; Stockert et al., 2016; Witte et al., 2000). Previous work suggests cortical damage from a stroke extends to remote regions beyond the frank lesion due to disruptions to structural pathways (Bonilha et al., 2014a), and damage to white matter tracts underlying the left temporal regions are commonly implicated in linguistic deficits (Baldo et al., 2013; Bonilha et al., 2014b; Butler et al., 2014; Dronkers et al., 2004; Fridriksson et al., 2013; Geva et al., 2012; Henseler et al., 2014; Ivanova et al., 2016). Consistent with these findings, we found that damage to ipsilesional structural pathways in the left temporal lobe (especially between superior temporal sulcus and middle temporal gyrus) predicts language impairments. Disruptions to interhemispheric connections also predicted linguistic deficits. Importantly, our findings contribute to a growing body of work investigating the effects of stroke lesions on global connectivity (Baldassarre et al., 2019; Griffis et al., 2019; Griffis et al., 2020; Hordacre et al., 2020; Sotelo et al., 2020; Wodeyar et al., 2020).

The main finding reveals unique contributions of rsFC to language performance after accounting for potentially confounding effects of structural damage. Others have shown an association between functional connectivity and comprehension where reduced connectivity in left fronto-parietal networks and right frontal regions is associated with comprehension deficits and increased connectivity in the same left fronto-parietal network is associated with recovery (Zhu et al., 2014). Similar to Zhu and colleagues, we show impaired functional connections in left fronto-parietal and temporoparietal regions are associated with reduced speech comprehension while increased coherence results in better performance. Unlike the previous study, however, our results control for potentially confounding effects of white matter damage in these regions and, therefore, suggest a distinct contribution of rsFC to language performance.

### Mechanisms Underlying Resting-State Functional Connectivity

Contributions of rsFC after regressing structural connectivity suggest that the rsFC identified here relies at least partially on indirect structural pathways. Neural networks are organized to minimize metabolic cost and for this reason they rely on direct structural pathways between two cortical nodes to maximize efficiency. In the context of a stroke-induced lesion, however, it seems likely that rsFC relies on alternate structural routes when direct connections are damaged. Indirect connectivity is less efficient and, presumably, weaker, which results in behavioral impairments, as evidenced by the associations with speech comprehension scores identified here. This finding corroborates recent work by Wodeyear and colleagues (2020) that found that for pairs of regions not directly anatomically connected, neural signals communicated by way of intermediate steps along an indirect anatomical path. Similarly, in an investigation of poststroke structural connectivity, Sotelo et al. (2020) identified relationships between indirect connectivity in regions remote from the lesion and motor impairment. In sum, our results contribute to a growing body of literature regarding the functionality of the brain poststroke. Our results and others (Sotelo et al., 2020; Wodeyar et al., 2020) show weak evidence in favor of a linear relationship between functional and structural connectivity after damage to structural connectivity networks. While the impact of a stroke on direct connections is well understood, such findings emphasize the broader impacts of a stroke lesion on global connectivity (Griffis et al., 2020; Wodeyar et al., 2020).

In the case of chronic stroke, functional connectivity networks that rely on indirect structural pathways likely reflect neural plasticity that occurs secondary to cortical damage. The dynamic properties of functional networks (Bullmore & Sporns, 2009; Park & Friston, 2013; Westlake et al., 2012) allow functional connectivity to reconfigure around underlying anatomical connections as a mechanism of neural plasticity (Hagmann et al., 2010; Hermundstad et al., 2014) Given that our data are from a cohort of chronic stroke participants, the functional connections identified here likely rely on indirect anatomical connections as a compensatory mechanism due to damage to direct structural pathways. This suggests poststroke aphasia recovery depends on the recruitment of intact but indirect structural pathways in residual brain tissue that is unaffected by necrosis or gliosis.

Functional connectivity depends on indirect structural pathways, even in undamaged neural networks (Sporns, 2011). Previous work has shown strong synchrony in BOLD signals exist between two cortical areas, even when direct structural pathways do not exist between those same two regions (Koch et al., 2002; Skudlarski et al., 2008; Vincent et al., 2007). Indirect connections can account for rsFC that is unexplained by direct connections (Honey et al., 2009); however, these connections are typically weaker than coherence from direct structural pathways (Goni et al., 2014). Future studies will need to include healthy controls to determine the anatomical connections that support ventral stream coherence, specifically, in speech comprehension in the absence of cortical damage (see *Limitations* section). Furthermore, it is important to acknowledge the dynamic and multifaceted processes of stroke recovery that contribute to global connectivity (see Dąbrowski et al., 2019, for a review). Regardless of the compensatory or preexisting nature of these connections, results from the current study suggests rsFC may be a robust biomarker to inform the neurobiology of aphasia. Here we show the predictive power of rsFC with regard to language performance and the relationship between rsFC and structural connectivity in the ipsilesional hemisphere.

## CLINICAL RELEVANCE AND FUTURE DIRECTIONS

### Measuring Neural Coherence

In the current study, we measured rsFC using fMRI. Measuring temporal coherence with alternative methodologies (i.e., EEG or MEG) will provide additional information regarding neural coherence. For example, EEG contributes information about synchronized activity between networks by measuring oscillatory changes with a high temporal resolution and offers information about frequency ranges of these oscillations. Better temporal resolution is needed as language processing occurs at much faster speeds than what we can measure with fMRI. Recent studies have utilized multimodal data (i.e., EEG paired with fMRI [Hordacre et al., 2020] and fNIRS [Arun et al., 2020]) to understand network connectivity and recovery dynamics in stroke patients. Incorporating such methodologies and considering multimodal approaches will continue to inform the relationship between brain structure and function to improve outcomes across behavioral domains. Each offers advantages to explain heterogeneity in patient performance poststroke, especially with regard to language.

### Modulating Neural Coherence

Identification of the neural mechanisms that underlie specific language impairments in aphasia is important to establish effective rehabilitation paradigms and improve prognostics. This work identifies neural synchrony that predicts speech comprehension and has the potential to inform neuro-rehabilitative paradigms such as noninvasive brain stimulation (NIBS). NIBS techniques such as transcranial direct current stimulation and transcranial alternating current stimulation (tACS) allow for the modulation of neural signals to improve functional connectivity; tACS, for example, delivers a low electrical current that entrains slow cortical oscillations and has the potential to modulate neural oscillations for enhanced behavioral outcomes (Riecke et al., 2015; Wilsch et al., 2018). Enhanced synchronization can improve network coherence and yield behavioral outcomes (Reinhart & Nguyen, 2019; Riecke et al., 2018). Future studies incorporating NIBS to modulate oscillations within the critical temporoparietal regions implicated here may improve behavioral outcomes, particularly speech comprehension, and improve our understanding of the neurobiology of aphasia to offer rehabilitation advances.

### Limitations

While this study provides evidence that impaired speech comprehension partially results from impaired neural synchrony, there are limitations that need to be addressed. First, this study relies on data from stroke patients and does not consider data from healthy controls. Without comparing to a normative sample, we cannot compare disordered rsFC to typical rsFC, precluding a comprehensive evaluation of poststroke changes in connectivity.

Second, our analysis used probabilistic tractography. While this method overcomes technical issues related to fiber crossing and complex fiber geometry, it is important to note that conventional probabilistic tractography (as applied in this study) does not report specific anatomical bundles of white matter, but identifies pathways between region of interest. Therefore, probabilistic tractography does not constrain the analyses based on specific white matter bundles and may map pathways that involve more than two regions if a pathway appears continuous. This is a limitation of tractography in general (deterministic or probabilistic).

Finally, we rely on behavioral subtests from the WAB-R as dependent variables (*Spontaneous Speech* and *Auditory Verbal Comprehension*). These assessments provide a coarse evaluation of language function for the purpose of determining aphasia severity and type. For example, the *Auditory Verbal Comprehension* subtest is a coarse, rather than a discrete measure of speech processing (i.e., varying sentence-level complexities via morphosyntactic structures). Coarse measures, such as the ones used here, may explain the lack of significant connections in the right hemisphere. Assessing bilaterally organized behaviors such as semantic retrieval and integration, executive function, and attention, as well as emotion and prosody recognition, may clarify interhemispheric coherence or right hemisphere functions. Similarly, the *Spontaneous Speech* subtest is a broad measure used to assess fluency and information content. Other limitations in the behavioral measures used here include clinician subjectivity, which may influence scores on the *Spontaneous Speech* subtest and may result in lower statistical power, perhaps partly explaining the lack of significant findings for speech production. Future studies may consider examining the associations between resting-state functional connectivity and discrete language measures.

### Conclusions

The current study contributes to an emerging body of research that suggests stroke-induced lesions have profound effects on neural networks. We control for potentially confounding effects of lesion volume and impaired structural connectivity to analyze rsFC in stroke. Our results show impaired speech comprehension poststroke is at least partially caused by loss of cortical synchrony in a left ventral stream network and is not simply reflective of localized necrosis or structural connectivity. We interpret these findings to suggest neural coherence (rsFC) offers predictive power for language processing even after accounting for cortical and white matter damage. This provides insight regarding rsFC as a biomarker of aphasia recovery and encourages implementation of multimodal neuroimaging to improve our understanding of the neurobiology of language.

## ACKNOWLEDGMENTS

The authors gratefully acknowledge participants and families for their participation in this study.

## AUTHOR CONTRIBUTIONS

Lynsey M. Keator: Data curation; Formal analysis; Methodology; Visualization; Writing – original draft; Writing – review & editing. Grigori Yourganov: Data curation; Formal analysis; Methodology; Writing – review & editing. Alexandra Basilakos: Data curation; Writing – review & editing. Argye Hillis: Data curation; Funding acquisition; Writing – review & editing. Gregory Hickok: Data curation; Funding acquisition; Writing – review & editing. Leonardo Bonilha: Data curation; Funding acquisition; Methodology; Writing – review & editing. Christopher Rorden: Data curation; Funding acquisition; Methodology; Visualization; Writing – review & editing. Julius Fridriksson: Conceptualization; Data curation; Formal analysis; Funding acquisition; Supervision; Writing – original draft; Writing – review & editing.

## FUNDING INFORMATION

Julius Fridriksson, National Institute on Deafness and Other Communication Disorders (https://dx.doi.org/10.13039/100000055), Award ID: P50 DC 014664.

## TECHNICAL TERMS

Structural connectivity: White matter fiber tracts between cortical brain regions.
Aphasia: Acquired language disorder that results from damage to language regions in the left hemisphere.
Resting-state functional connectivity: A measure of functional connectivity that reflects temporal coherence of BOLD signal fluctuations between gray matter regions.
BOLD signal: Blood oxygen level–dependent signals.
Dual-stream model: Theoretical model of language processing that posits a unilateral dorsal stream for language production and bilateral ventral stream for comprehension.
Western Aphasia Battery–Revised: An assessment of language function (i.e., fluency, word finding, comprehension) to determine aphasia type and level of severity.
Auditory verbal comprehension: Measure from the WAB-R to assess understanding of spoken language (i.e., yes/no questions, auditory word recognition, comments).
*Spontaneous Speech* subtest: Measure from the WAB-R to assess expressive language (i.e., response to open-ended questions and verbal description of a picture).
AICHA atlas: Brain atlas that includes 192 homotopic region pairs (122 gyral, 50 sulcal, and 20 gray nuclei).
Lesion-symptom mapping: An analysis used to determine the relationship between brain damage and behavior.
Probabilistic tractography: An analysis used to calculate streamline propagation and infer the strength of structural connectivity.

## References

[bib1] Adachi, Y., Osada, T., Sporns, O., Watanabe, T., Matsui, T., Miyamoto, K., & Miyashita, Y. (2012). Functional connectivity between anatomically unconnected areas is shaped by collective network-level effects in the macaque cortex. Cerebral Cortex, 22(7), 1586–1592. https://doi.org/10.1093/cercor/bhr234, PubMed: 21893683 21893683 10.1093/cercor/bhr234

[bib2] Arun, K. M., Smitha, K. A., Sylaja, P. N., & Kesavadas, C. (2020). Identifying resting-state functional connectivity changes in the motor cortex using fNIRS during recovery from stroke. Brain Topography, 33(6), 710–719. https://doi.org/10.1007/s10548-020-00785-2, PubMed: 32685998 32685998 10.1007/s10548-020-00785-2

[bib3] Baldassarre, A., Ramsey, L., Siegel, J., Shulman, G., & Corbetta, M. (2016). Brain connectivity and neurological disorders after stroke. Current Opinion in Neurology, 29(6), 706–713. https://doi.org/10.1097/WCO.0000000000000396, PubMed: 27749394 27749394 10.1097/WCO.0000000000000396 PMC5682022

[bib4] Baldassarre, A., Metcalf, N. V., Shulman, G. L., & Corbetta, M. (2019). Brain networks’ functional connectivity separates aphasic deficits in stroke. Neurology, 92(2), E125–E135. https://doi.org/10.1212/WNL.0000000000006738, PubMed: 30518552 30518552 10.1212/WNL.0000000000006738 PMC6340343

[bib5] Baldo, J. V., Arévalo, A., Patterson, J. P., & Dronkers, N. F. (2013). Grey and white matter correlates of picture naming: Evidence from a voxel-based lesion analysis of the Boston Naming Test. Cortex, 49(3), 658–667. https://doi.org/10.1016/j.cortex.2012.03.001, PubMed: 22482693 22482693 10.1016/j.cortex.2012.03.001 PMC3613759

[bib6] Behrens, T. E. J., Berg, H. J., Jbabdi, S., Rushworth, M. F. S., & Woolrich, M. W. (2007). Probabilistic diffusion tractography with multiple fibre orientations: What can we gain? NeuroImage, 34(1), 144–155. https://doi.org/10.1016/j.neuroimage.2006.09.018, PubMed: 17070705 17070705 10.1016/j.neuroimage.2006.09.018 PMC7116582

[bib7] Biswal, B., Yetkin, F. Z., Haughton, V. M., & Hyde, J. (1995). Functional connectivity in the motor cortex of resting human brain using echo-planar MRI. Magnetic Resonance Medicine, 34(4), 537–541. https://doi.org/10.1002/mrm.1910340409, PubMed: 8524021 10.1002/mrm.1910340409 8524021

[bib8] Bonilha, L., Gleichgerrcht, E., Fridriksson, J., Breedlove, J. L., Rorden, C., Nesland, T., … Focke, N. K. (2015). Reproducibility of the structural brain connectome derived from diffusion tensor imaging. PLoS ONE, 10(9), 1–17. https://doi.org/10.1371/journal.pone.0135247, PubMed: 26332788 10.1371/journal.pone.0135247 PMC4557836 26332788

[bib9] Bonilha, L., Nesland, T., Rorden, C., Fillmore, P., Ratnayake, R. P., & Fridriksson, J. (2014a). Mapping remote subcortical ramifications of injury after ischemic strokes. Behavioural Neurology, 2014. https://doi.org/10.1155/2014/215380, PubMed: 24868120 10.1155/2014/215380 PMC4017848 24868120

[bib10] Bonilha, L., Rorden, C., & Fridriksson, J. (2014b). Assessing the clinical impact of residual cortical disconnection after ischemic strokes. Stroke, 45(4), 988–993. https://doi.org/10.1161/STROKEAHA.113.004137, PubMed: 24619391 24619391 10.1161/STROKEAHA.113.004137 PMC4504196

[bib11] Bullmore, E., & Sporns, O. (2009). Complex brain networks: Graph theoretical analysis of structural and functional systems. Nature Reviews Neuroscience, 10(3), 186–198. https://doi.org/10.1038/nrn2575, PubMed: 19190637 19190637 10.1038/nrn2575

[bib80] Brott, T., Adams, H. P., Jr., (1989). Measurements of acute cerebral infarction: A clinical examination scale. Stroke, 20(7), 864–870. https://doi.org/10.1161/01.str.20.7.864, PubMed: 2749846 2749846 10.1161/01.str.20.7.864

[bib12] Butler, R. A., Ralph, M. A. L., & Woollams, A. M. (2014). Capturing multidimensionality in stroke aphasia: Mapping principal behavioural components to neural structures. Brain, 137(12), 3248–3266. https://doi.org/10.1093/brain/awu286, PubMed: 25348632 25348632 10.1093/brain/awu286 PMC4240295

[bib13] Carrera, E., & Tononi, G. (2014). Diaschisis: Past, present, future. Brain, 137(9), 2408–2422. https://doi.org/10.1093/brain/awu101, PubMed: 24871646 24871646 10.1093/brain/awu101

[bib14] Carter, A. R., Astafiev, S. V., Lang, C. E., Connor, L. T., Rengachary, J., Strube, M. J., … Corbetta, M. (2010). Resting interhemispheric functional magnetic resonance imaging connectivity predicts performance after stroke. Annals of Neurology, 67(3), 365–375. https://doi.org/10.1002/ana.21905, PubMed: 20373348 20373348 10.1002/ana.21905 PMC2927671

[bib15] Chiang, S., Stern, J. M., Engel, J., & Haneef, Z. (2015). Structural-functional coupling changes in temporal lobe epilepsy. Brain Research Reviews, 1616, 45–57. https://doi.org/10.1016/j.brainres.2015.04.052, PubMed: 25960346 10.1016/j.brainres.2015.04.052 PMC4892844 25960346

[bib16] Corbetta, M., Kincade, M. J., Lewis, C., Snyder, A. Z., & Sapir, A. (2005). Neural basis and recovery of spatial attention deficits in spatial neglect. Nature Neuroscience, 8(11), 1603–1610. https://doi.org/10.1038/nn1574, PubMed: 16234807 16234807 10.1038/nn1574

[bib17] Corbetta, M., Siegel, J. S., & Shulman, G. L. (2018). On the low dimensionality of behavioral deficits and alterations of brain network connectivity after focal injury. Cortex, 107, 229–237. https://doi.org/10.1016/j.cortex.2017.12.017, PubMed: 29357980 29357980 10.1016/j.cortex.2017.12.017 PMC6028302

[bib18] Cramer, S. C. (2008). Repairing the human brain after stroke: I. Mechanisms of spontaneous recovery. Annals of Neurology, 63(3), 272–287. https://doi.org/10.1002/ana.21393, PubMed: 18383072 18383072 10.1002/ana.21393

[bib19] Dąbrowski, J., Czajka, A., Zielińska-Turek, J., Jaroszyński, J., Furtak-Niczyporuk, M., Mela, A., … Zabel, M. (2019). Brain functional reserve in the context of neuroplasticity after stroke. Neural Plasticity, 2019. https://doi.org/10.1155/2019/9708905, PubMed: 30936915 10.1155/2019/9708905 PMC6415310 30936915

[bib20] Damoiseaux, J. S., & Greicius, M. D. (2009). Greater than the sum of its parts: A review of studies combining structural connectivity and resting-state functional connectivity. Brain Structure and Function, 213(6), 525–533. https://doi.org/10.1007/s00429-009-0208-6, PubMed: 19565262 19565262 10.1007/s00429-009-0208-6

[bib21] Dronkers, N. F., Wilkins, D. P., Van Valin, R. D., Redfern, B. B., & Jaeger, J. J. (2004). Lesion analysis of the brain areas involved in language comprehension. Cognition, 92(1–2), 145–177. https://doi.org/10.1016/j.cognition.2003.11.002, PubMed: 15037129 15037129 10.1016/j.cognition.2003.11.002

[bib22] Faria, A. V., Joel, S. E., Zhang, Y., Oishi, K., van Zjil, P. C. M., Miller, M. I., … Mori, S. (2012). Atlas-based analysis of resting-state functional connectivity: Evaluation for reproducibility and multi-modal anatomy-function correlation studies. NeuroImage, 61(3), 613–621. https://doi.org/10.1016/j.neuroimage.2012.03.078, PubMed: 22498656 22498656 10.1016/j.neuroimage.2012.03.078 PMC3358461

[bib23] Fox, M. D., & Raichle, M. E. (2007). Spontaneous fluctuations in brain activity observed with functional magnetic resonance imaging. Nature Reviews Neuroscience, 8, 700–711. https://doi.org/10.1038/nrn2201, PubMed: 17704812 17704812 10.1038/nrn2201

[bib24] Fridriksson, J., Guo, D., Fillmore, P., Holland, A., & Rorden, C. (2013). Damage to the anterior arcuate fasciculus predicts non-fluent speech production in aphasia. Brain, 136(11), 3451–3460. https://doi.org/10.1093/brain/awt267, PubMed: 24131592 24131592 10.1093/brain/awt267 PMC3808690

[bib25] Fridriksson, J., Yourganov, G., Bonilha, L., Basilakos, A., Den Ouden, D.-B., & Rorden, C. (2016). Revealing the dual streams of speech processing. Proceedings of the National Academy of Sciences of the United States of America, 113(52), 15108–15113. https://doi.org/10.1073/pnas.1614038114, PubMed: 27956600 27956600 10.1073/pnas.1614038114 PMC5206517

[bib26] Friederici, A. D., & Wartenburger, I. (2010). Language and brain. WIREs Cognitive Science, 1, 150–159. https://doi.org/10.1002/wcs.9, PubMed: 26271230 26271230 10.1002/wcs.9

[bib27] Geva, S., Baron, J. C., Jones, P. S., Price, C. J., & Warburton, E. A. (2012). A comparison of VLSM and VBM in a cohort of patients with post-stroke aphasia. NeuroImage: Clinical, 1(1), 37–47. https://doi.org/10.1016/j.nicl.2012.08.003, PubMed: 24179735 24179735 10.1016/j.nicl.2012.08.003 PMC3757730

[bib28] Gleichgerrcht, E., Fridriksson, J., Rorden, C., & Bonilha, L. (2017). Connectome-based lesion-symptom mapping (CLSM): A novel approach to map neurological function. NeuroImage: Clinical, 16(April), 461–467. https://doi.org/10.1016/j.nicl.2017.08.018, PubMed: 28884073 28884073 10.1016/j.nicl.2017.08.018 PMC5581860

[bib29] Goni, J., Van Den Heuvel, M. P., Avena-Koenigsberger, A., De Mendizabal, N. V., Betzel, R. F., Griffa, A., … Sporns, O. (2014). Resting-brain functional connectivity predicted by analytic measures of network communication. Proceedings of the National Academy of Sciences of the United States of America, 111(2), 833–838. https://doi.org/10.1073/pnas.1315529111, PubMed: 24379387 24379387 10.1073/pnas.1315529111 PMC3896172

[bib30] Griffis, J. C., Metcalf, N. V., Corbetta, M., & Shulman, G. L. (2019). Structural disconnections explain brain network dysfunction after stroke. Cell Reports, 28(10), 2527–2540. https://doi.org/10.1016/j.celrep.2019.07.100, PubMed: 31484066 31484066 10.1016/j.celrep.2019.07.100 PMC7032047

[bib31] Griffis, J. C., Metcalf, N. V., Corbetta, M., & Shulman, G. L. (2020). Damage to the shortest structural paths between brain regions is associated with disruptions of resting-state functional connectivity after stroke. NeuroImage, 210(January). https://doi.org/10.1016/j.neuroimage.2020.116589, PubMed: 32007498 10.1016/j.neuroimage.2020.116589 PMC7061444 32007498

[bib32] Griffis, J. C., Nenert, R., Allendorfer, J. B., & Szaflarski, J. P. (2017). Damage to white matter bottlenecks contributes to language impairments after left hemispheric stroke. NeuroImage: Clinical, 14, 552–565. https://doi.org/10.1016/j.nicl.2017.02.019, PubMed: 28337410 28337410 10.1016/j.nicl.2017.02.019 PMC5350568

[bib33] Hagmann, P., Sporns, O., Madan, N., Cammoun, L., Pienaar, R., Wedeen, V. J., … Grant, P. E. (2010). White matter maturation reshapes structural connectivity in the late developing human brain. Proceedings of the National Academy of Sciences of the United States of America, 107(44), 19067–19072. https://doi.org/10.1073/pnas.1009073107, PubMed: 20956328 20956328 10.1073/pnas.1009073107 PMC2973853

[bib34] He, B. J., Snyder, A. Z., Vincent, J. L., Epstein, A., Shulman, G. L., & Corbetta, M. (2007). Breakdown of functional connectivity in frontoparietal networks underlies behavioral deficits in spatial neglect. Neuron, 53(6), 905–918. https://doi.org/10.1016/j.neuron.2007.02.013, PubMed: 17359924 17359924 10.1016/j.neuron.2007.02.013

[bib35] Henseler, I., Regenbrecht, F., & Obrig, H. (2014). Lesion correlates of patholinguistic profiles in chronic aphasia: Comparisons of syndrome-, modality- and symptom-level assessment. Brain, 137(3), 918–930. https://doi.org/10.1093/brain/awt374, PubMed: 24525451 24525451 10.1093/brain/awt374

[bib36] Hermundstad, A. M., Brown, K. S., Bassett, D. S., Aminoff, E. M., Frithsen, A., Johnson, A., … Carlson, J. M. (2014). Structurally-constrained relationships between cognitive states in the human brain. PLoS Computational Biology, 10(5). https://doi.org/10.1371/journal.pcbi.1003591, PubMed: 24830758 10.1371/journal.pcbi.1003591 PMC4022461 24830758

[bib37] Hickok, G., & Poeppel, D. (2004). Dorsal and ventral streams: A framework for understanding aspects of the functional anatomy of language. Cognition, 92(1–2), 67–99. https://doi.org/10.1016/j.cognition.2003.10.011, PubMed: 15037127 15037127 10.1016/j.cognition.2003.10.011

[bib38] Hickok, G., & Poeppel, D. (2007). The cortical organization of speech processing. Nature Reviews Neuroscience, 8(May), 393–402. https://doi.org/10.1038/nrn2113, PubMed: 17431404 17431404 10.1038/nrn2113

[bib39] Honey, C. J., Sporns, O., Cammoun, L., Gigandet, X., Thiran, J. P., Meuli, R., & Hagmann, P. (2009). Predicting human resting-state functional connectivity from structural connectivity. Proceedings of the National Academy of Sciences of the United States of America, 106(6), 2035–2040. https://doi.org/10.1073/pnas.0811168106, PubMed: 19188601 19188601 10.1073/pnas.0811168106 PMC2634800

[bib40] Honey, C. J., & Sporns, O. (2008). Dynamical consequences of lesions in cortical networks. Human Brain Mapping, 29(7), 802–809. https://doi.org/10.1002/hbm.20579, PubMed: 18438885 18438885 10.1002/hbm.20579 PMC6870962

[bib41] Hope, T. M. H., Leff, A. P., Prejawa, S., Bruce, R., Haigh, Z., Lim, L., … Price, C. J. (2017). Right hemisphere structural adaptation and changing language skills years after left hemisphere stroke. Brain, 140, 1718–1728. https://doi.org/10.1093/brain/awx086, PubMed: 28444235 28444235 10.1093/brain/awx086 PMC5445256

[bib42] Hordacre, B., Goldsworthy, M. R., Welsby, E., Graetz, L., Ballinger, S., & Hillier, S. (2020). Resting state functional connectivity is associated with motor pathway integrity and upper-limb behavior in chronic stroke. Neurorehabilitation and Neural Repair, 34(6), 547–557. https://doi.org/10.1177/1545968320921824, PubMed: 32436426 32436426 10.1177/1545968320921824

[bib43] Ivanova, M. V., Isaev, D. Y., Dragoy, O. V., Akinina, Y. S., Petrushevskiy, A. G., Fedina, O. N., … Dronkers, N. F. (2016). Diffusion-tensor imaging of major white matter tracts and their role in language processing in aphasia. Cortex, 85, 165–181. https://doi.org/10.1016/j.cortex.2016.04.019, PubMed: 27289586 27289586 10.1016/j.cortex.2016.04.019

[bib44] Joliot, M., Jobard, G., Naveau, M., Delcroix, N., Petit, L., Zago, L., … Tzourio-Mazoyer, N. (2015). AICHA: An atlas of intrinsic connectivity of homotopic areas. Journal of Neuroscience Methods, 254, 46–59. https://doi.org/10.1016/j.jneumeth.2015.07.013, PubMed: 26213217 26213217 10.1016/j.jneumeth.2015.07.013

[bib46] Kertesz, A. (2007). Western Aphasia Battery-Revised. San Antonio, TX: Psychological Corporation. 10.1037/t15168-000

[bib47] Klingbeil, J., Wawrzyniak, M., Stockert, A., & Saur, D. (2019). Resting-state functional connectivity: An emerging method for the study of language networks in post-stroke aphasia. Brain and Cognition, 131(August 2017), 22–33. https://doi.org/10.1016/j.bandc.2017.08.005, PubMed: 28865994 28865994 10.1016/j.bandc.2017.08.005

[bib48] Koch, M. A., Norris, D. G., & Hund-Georgiadis, M. (2002). An investigation of functional and anatomical connectivity using magnetic resonance imaging. NeuroImage, 16(1), 241–250. https://doi.org/10.1006/nimg.2001.1052, PubMed: 11969331 11969331 10.1006/nimg.2001.1052

[bib49] Kreisel, S. H., Bazner, H., & Hennerici, M. (2006). Pathophysiology of stroke rehabilitation: temporal aspects of neuro-functional recovery. Cerebrovascular Diseases, 21(1–2), 6–17. https://doi.org/10.1159/000089588, PubMed: 16282685 16282685 10.1159/000089588

[bib50] Kümmerer, D., Hartwigsen, G., Kellmeyer, P., Glauche, V., Mader, I., Klöppel, S., … Saur, D. (2013). Damage to ventral and dorsal language pathways in acute aphasia. Brain, 136(2), 619–629. https://doi.org/10.1093/brain/aws354, PubMed: 23378217 23378217 10.1093/brain/aws354 PMC3572927

[bib51] Min, Y.-S., Park, J. W., Park, E., Kim, A.-R., Cha, H., Gwak, D.-W., … Jung, T.-D. (2020). Interhemispheric functional connectivity in the primary motor cortex assessed by resting-state functional magnetic resonance imaging aids long-term recovery prediction among subacute stroke patients with severe hand weakness. Journal of Clinical Medicine, 9(4), 975. https://doi.org/10.3390/jcm9040975, PubMed: 32244596 10.3390/jcm9040975 PMC7230262 32244596

[bib52] Miŝic, B., Betzel, R. F., De Reus, M. A., Van den Heuvel, M. P., Berman, M. G., McIntosh, A. R., & Sporns, O. (2016). Network-level structure-function relationships in human neocortex. Cerebral Cortex, 26(7), 3285–3296. https://doi.org/10.1093/cercor/bhw089, PubMed: 27102654 27102654 10.1093/cercor/bhw089 PMC4898678

[bib53] Nudo, R. J., & Friel, K. (1999). Cortical plasticity after stroke: Implications for rehabilitation. Review of Neurology (Paris), 155(9), 713–717. 10528355

[bib54] Park, H. J., & Friston, K. (2013). Structural and functional brain networks: From connections to cognition. Science, 342(6158). https://doi.org/10.1126/science.1238411, PubMed: 24179229 10.1126/science.1238411 24179229

[bib55] Park, C., Chang, W. H., Ohn, S. H., Kim, S. T., Bang, O. Y., Pascual-Leone, A., & Kim, Y. (2011). Longitudinal changes of resting-state functional connectivity during motor recovery after stroke. Stroke, 42, 1357–1362. https://doi.org/10.1161/STROKEAHA.110.596155, PubMed: 21441147 21441147 10.1161/STROKEAHA.110.596155 PMC3589816

[bib56] Reinhart, R. M. G., & Nguyen, J. A. (2019). Working memory revived in older adults by synchronizing rhythmic brain circuits. Nature Neuroscience, 22(5), 820–827. https://doi.org/10.1038/s41593-019-0371-x, PubMed: 30962628 30962628 10.1038/s41593-019-0371-x PMC6486414

[bib57] Riecke, L., Formisano, E., Herrmann, C. S., & Sack, A. T. (2015). 4-Hz transcranial alternating current stimulation phase modulates hearing. Brain Stimulation, 8(4), 777–783. https://doi.org/10.1016/j.brs.2015.04.004, PubMed: 25981160 25981160 10.1016/j.brs.2015.04.004

[bib58] Riecke, L., Formisano, E., Sorger, B., Bas¸kent, D., & Gaudrain, E. (2018). Neural entrainment to speech modulates speech intelligibility. Current Biology, 28(2), 161–169. https://doi.org/10.1016/j.cub.2017.11.033, PubMed: 29290557 29290557 10.1016/j.cub.2017.11.033

[bib59] Rorden, C., Bonilha, L., Fridriksson, J., Bender, B., & Karnath, O. (2013). Age-specific CT and MRI templates for spatial normalization. NeuroImage, 61(4), 957–965. https://doi.org/10.1016/j.neuroimage.2012.03.020, PubMed: 22440645 10.1016/j.neuroimage.2012.03.020 PMC3376197 22440645

[bib60] Rorden, C., & Brett, M. (2000). Steriotaxic display of brain lesions. Behavioural Neurology, 12(4), 191–200. https://doi.org/10.1155/2000/421719, PubMed: 11568431 11568431 10.1155/2000/421719

[bib61] Salvalaggio, A., De Filippo De Grazia, M., Zorzi, M., Thiebaut de Schotten, M., & Corbetta, M. (2020). Post-stroke deficit prediction from lesion and indirect structural and functional disconnection. Brain: A Journal of Neurology, 143(7), 2173–2188. https://doi.org/10.1093/brain/awaa156, PubMed: 32572442 32572442 10.1093/brain/awaa156 PMC7363494

[bib62] Saur, D., Kreher, B. W., Schnell, S., Kümmerer, D., Kellmeyer, P., Vry, M.-S., … Weiller, C. (2008). Ventral and dorsal pathways for language. Proceedings of the National Academy of Sciences of the United States of America, 105(46), 18035–18040. https://doi.org/10.1073/pnas.0805234105, PubMed: 19004769 19004769 10.1073/pnas.0805234105 PMC2584675

[bib63] Siegel, J. S., Ramsey, L. E., Snyder, A. Z., Metcalf, N. V., Chacko, R. V., Weinberger, K., … Corbetta, M. (2016). Disruptions of network connectivity predict impairment in multiple behavioral domains after stroke. Proceedings of the National Academy of Sciences of Sciences of the United States of America, 113(30), E4367–E4376. https://doi.org/10.1073/pnas.1521083113, PubMed: 27402738 10.1073/pnas.1521083113 PMC4968743 27402738

[bib64] Skudlarski, P., Jagannathan, K., Calhoun, V. D., Hampson, M., Skudlarska, B. A., & Pearlson, G. (2008). Measuring brain connectivity: Diffusion tensor imaging validates resting state temporal correlations. NeuroImage, 43(3), 554–561. https://doi.org/10.1016/j.neuroimage.2008.07.063, PubMed: 18771736 18771736 10.1016/j.neuroimage.2008.07.063 PMC4361080

[bib65] Sotelo, M. R., Kalinosky, B. T., Goodfriend, K., Hyngstrom, A. S., & Schmit, B. D. (2020). Indirect structural connectivity identifies changes in brain networks after stroke. Brain Connectivity, 10(8), 399–410. https://doi.org/10.1089/brain.2019.0725, PubMed: 32731752 32731752 10.1089/brain.2019.0725

[bib66] Sporns, O. (2011). Networks of the brain. Cambridge, MA: MIT Press. 10.7551/mitpress/8476.001.0001

[bib67] Stockert, A., Kümmerer, D., & Saur, D. (2016). Insights into early language recovery: From basic principles to practical applications. Aphasiology, 30(5), 517–541. 10.1080/02687038.2015.1119796

[bib68] Tang, C., Zhao, Z., Chen, C., Zheng, X., Sun, F., Zhang, X., … Jia, J. (2016). Decreased functional connectivity of homotopic brain regions in chronic stroke patients: A resting state fMRI study. PLoS ONE, 11(4), 1–13. https://doi.org/10.1371/journal.pone.0152875, PubMed: 27074031 10.1371/journal.pone.0152875 PMC4830618 27074031

[bib69] Vincent, J. L., Patel, G. H., Fox, M. D., Snyder, A. Z., Baker, J. T., Van Essen, D. C., … Raichle, M. E. (2007). Intrinsic functional architecture in the anaesthetized monkey brain. Nature, 447(7140), 83–86. https://doi.org/10.1038/nature05758, PubMed: 17476267 17476267 10.1038/nature05758

[bib70] Westlake, K., Hinkley, L., Bucci, M., Guggisberg, A., Findlay, A., Byl, N., Henry, R., & Nagarajan, S. (2012). Resting state alpha-band functional connectivity and recovery after stroke. Experimental Neurology, 237(1), 160–169. https://doi.org/10.1016/j.expneurol.2012.06.020, PubMed: 22750324 22750324 10.1016/j.expneurol.2012.06.020 PMC3646713

[bib71] Wilsch, A., Neuling, T., Obleser, J., & Herrmann, C. S. (2018). Transcranial alternating current stimulation with speech envelopes modulates speech comprehension. NeuroImage, 172(July 2017), 766–774. https://doi.org/10.1016/j.neuroimage.2018.01.038, PubMed: 29355765 29355765 10.1016/j.neuroimage.2018.01.038

[bib72] Witte, O. W., Bidmon, H., Schiene, K., Redecker, C., & Hagemann, G. (2000). Functional differentiation of multiple perilesional zones after focal cerebral ischemia. Journal of Cerebral Blood Flow and Metabolism, 20, 1149–1165. https://doi.org/10.1097/00004647-200008000-00001, PubMed: 10950376 10950376 10.1097/00004647-200008000-00001

[bib73] Wodeyar, A., Cassidy, J. M., Cramer, S. C., & Srinivasan, R. (2020). Damage to the structural connectome reflected in resting-state fMRI functional connectivity. Network Neuroscience, 1–22. https://doi.org/10.1162/netn_a_00160, PubMed: 33409436 33409436 10.1162/netn_a_00160 PMC7781612

[bib74] Yourganov, G., Fridriksson, J., Rorden, C., Gleichgerrcht, E., & Bonilha, L. (2016). Multivariate connectome-based symptom mapping in post-stroke patients: Networks supporting language and speech. Journal of Neuroscience, 36(25), 6668–6679. https://doi.org/10.1523/JNEUROSCI.4396-15.2016, PubMed: 27335399 27335399 10.1523/JNEUROSCI.4396-15.2016 PMC4916245

[bib75] Yourganov, G., Fridriksson, J., Stark, B., & Rorden, C. (2018). Removal of artifacts from resting-state fMRI data in stroke. NeuroImage: Clinical, 17(September 2017), 297–305. https://doi.org/10.1016/j.nicl.2017.10.027, PubMed: 29527477 29527477 10.1016/j.nicl.2017.10.027 PMC5842649

[bib76] Zhang, J., Zhang, Y., Wang, L., Sang, L., Yang, J., Yan, R., … Qiu, M. (2017). Disrupted structural and functional connectivity networks in ischemic stroke patients. Neuroscience, 364, 212–225. https://doi.org/10.1016/j.neuroscience.2017.09.009, PubMed: 28918259 28918259 10.1016/j.neuroscience.2017.09.009

[bib77] Zhang, Z., Liao, W., Chen, H., Mantini, D., Ding, J. R., Xu, Q., … Lu, G. (2011). Altered functional-structural coupling of large-scale brain networks in idiopathic generalized epilepsy. Brain, 134(10), 2912–2928. https://doi.org/10.1093/brain/awr223, PubMed: 21975588 21975588 10.1093/brain/awr223

[bib78] Zhu, D., Chang, J., Freeman, S., Tan, Z., Xiao, J., Gao, Y., & Kong, J. (2014). Changes of functional connectivity in the left frontoparietal network following aphasic stroke. Frontiers in Behavioral Neuroscience, 8(May), 1–10. https://doi.org/10.3389/fnbeh.2014.00167, PubMed: 24860452 24860452 10.3389/fnbeh.2014.00167 PMC4026698

